# Giant gastrointestinal stromal tumor of the mediastinum associated with an esophageal hiatal hernia and chest discomfort: a case report

**DOI:** 10.1186/s40792-018-0553-x

**Published:** 2018-12-13

**Authors:** Ryosuke Fujisawa, Yuji Akiyama, Takeshi Iwaya, Fumitaka Endo, Haruka Nikai, Shigeaki Baba, Takehiro Chiba, Toshimoto Kimura, Takeshi Takahara, Koki Otsuka, Hiroyuki Nitta, Masaru Mizuno, Keisuke Koeda, Akira Sasaki

**Affiliations:** 1 Department of Surgery, Iwate Medical University School of Medicine, Iwate, Japan; 2 Department of Medical Safety Science, Iwate Medical University School of Medicine, Iwate, Japan

**Keywords:** Gastric gastrointestinal stromal tumor, Giant gastrointestinal stromal tumor, Mediastinal tumor, Hiatal hernia

## Abstract

**Background:**

Gastrointestinal stromal tumors (GISTs) grow relatively slowly and without specific symptoms; therefore, they are typically incidental findings. We report a rare gastric GIST in the mediastinum associated with chest discomfort and an esophageal hiatal hernia.

**Case presentation:**

An 81-year-old woman with chest discomfort was admitted to the hospital, where barium esophagography showed a sliding esophageal hiatal hernia and a tumor of the lower esophagus and gastric wall. Esophagogastroscopy confirmed the presence of a huge submucosal tumor that extended from the lower esophagus to the gastric fundus. According to computed tomography, the mediastinal mass measured 12.7 cm and had heterogeneous low-density areas. A submucosal gastric tumor, which we suspected to be a GIST, was diagnosed in association with an esophageal hiatal hernia. Using thoracolaparotomy, we performed a total gastrectomy, a lower esophagectomy, and a Roux-en-Y reconstruction with the jejunum. The presumptive diagnosis was confirmed through immunohistochemical examination; immunostaining yielded results positive for CD34 and c-kit. The patient was discharged from the hospital 13 days after surgery with no complications and remained disease-free at follow-up 24 months after surgery.

**Conclusions:**

GIST should be considered in the differential diagnosis of tumors growing in the mediastinum.

## Background

Gastrointestinal stromal tumors (GISTs) are the most common mesenchymal tumors of the gastrointestinal tract [1], although they account for only 1–3% of all gastrointestinal tumors [2]. They grow relatively slowly, producing no specific symptoms, and 60–70% originate from the stomach [3–5]. Consequently, GISTs tend to be found incidentally [3, 6]. We report a rare gastric GIST in the mediastinum that was associated with chest discomfort and an esophageal hiatal hernia.

## Case presentation

**Fig. 1:**
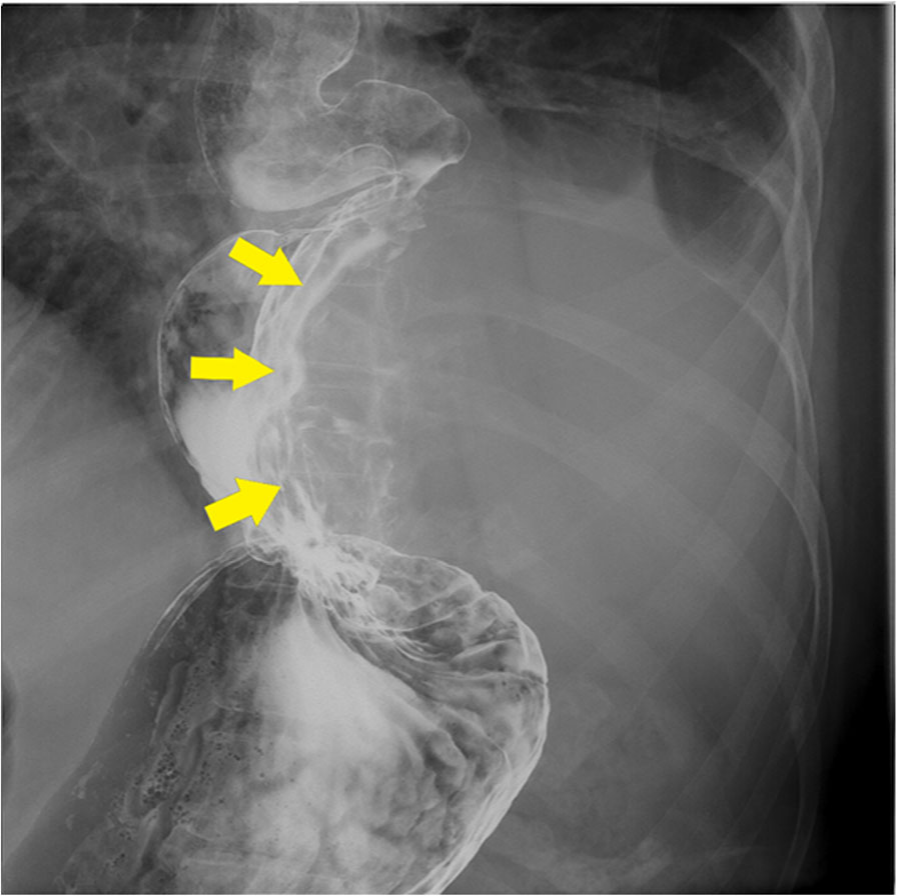
Barium esophagogram showing a sliding esophageal hiatal hernia and defects of the lower esophagus and gastric wall caused by a huge tumor

An 81-year-old woman was admitted with chest discomfort. She had a history of appendectomy, hypertension, colon polyps, and osteoporosis. Physical examination revealed no tenderness and no palpable mass in the abdomen. Laboratory investigation yielded unremarkable results, and the values for hemoglobin and tumor markers (including carcinoembryonic antigen and CA19-9) were normal. Barium esophagography, ordered because of the clinical findings, revealed a sliding esophageal hiatal hernia associated with a defect of the lower esophagus and the gastric wall that was caused by a huge tumor (Fig. 1).

**Fig. 2:**
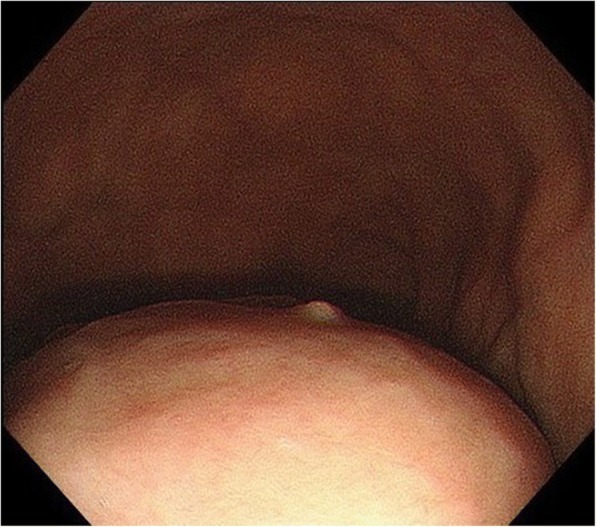
Esophagogastroscopy showing a huge, hard, elastic, submucosal tumor that extends from the lower esophagus to the gastric fundus

**Fig. 3:**
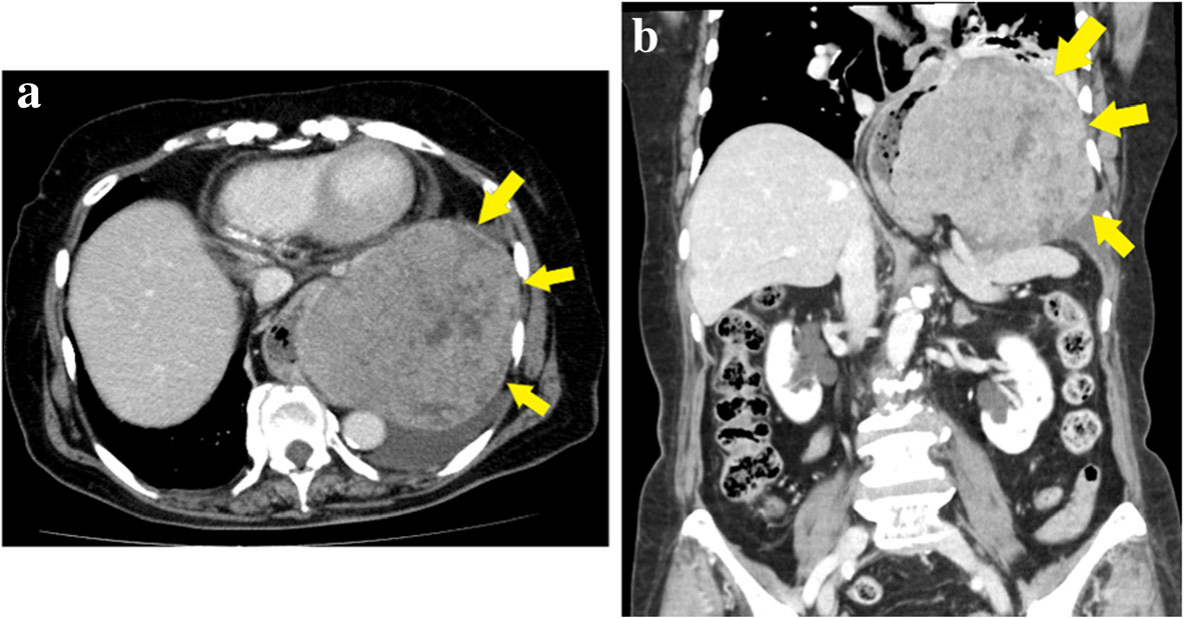
Chest and abdominal computed tomographic scans showing a 13-cm mass in the mediastinum. **a** Axial view. **b** Coronal view

Esophagogastroscopy revealed a severe hiatal hernia and a huge, hard, elastic submucosal tumor, extending from the lower esophagus to the gastric fundus (Fig. 2). Chest and abdominal computed tomography (CT) showed a 12.7-cm mass in the mediastinum; the mass was solid with some low-density areas (Fig. 3a, b). In addition, CT revealed that the mass was continuous with the gastric wall, and its border with the esophagus was clear. Therefore, we determined that the mass was a tumor that had arisen from the stomach. The diagnosis was of a submucosal tumor of the stomach, complicated by an esophageal hiatal hernia. On the basis of these findings, we opted for surgical resection.

**Fig. 4:**
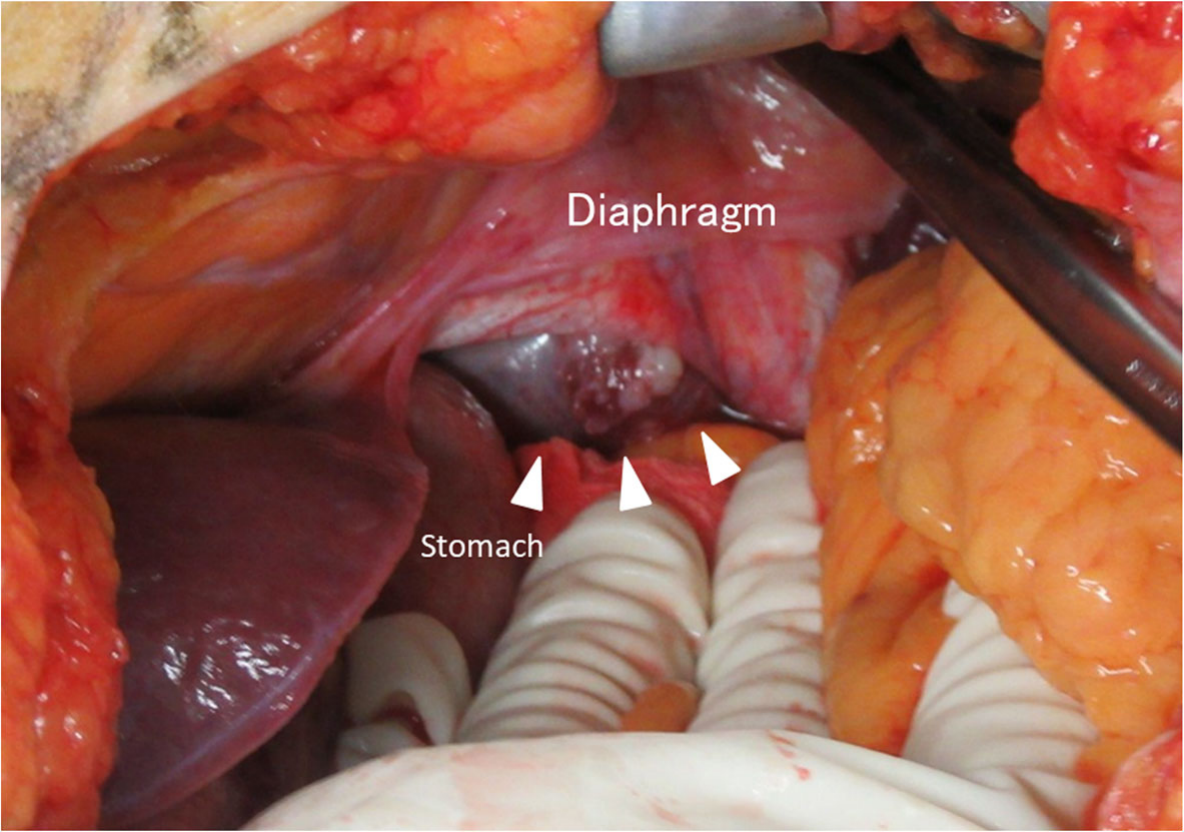
Intraoperative findings. The tumor (arrowheads) can be seen arising from the stomach wall and extending to greater curvature of the gastric fundus. We approached the mediastinum by dissection of the diaphragm

In laparotomy, we first approached the tumor by dissection of the diaphragm (Fig. 4). This revealed a huge tumor that arose from the stomach wall and adhered to the lower lobe of the left lung, the mediastinal pleura, the diaphragm, and the esophagus. Because further tumor dissection was difficult, we instead performed an additional thoracotomy through the left sixth intercostal space. Next, taking care to avoid damaging the outer membrane, we performed a total gastrectomy, a lower esophagectomy, and a Roux-en-Y jejunal reconstruction. The surgical time was 357 min, and the total blood loss was 292 mL.

**Fig. 5:**
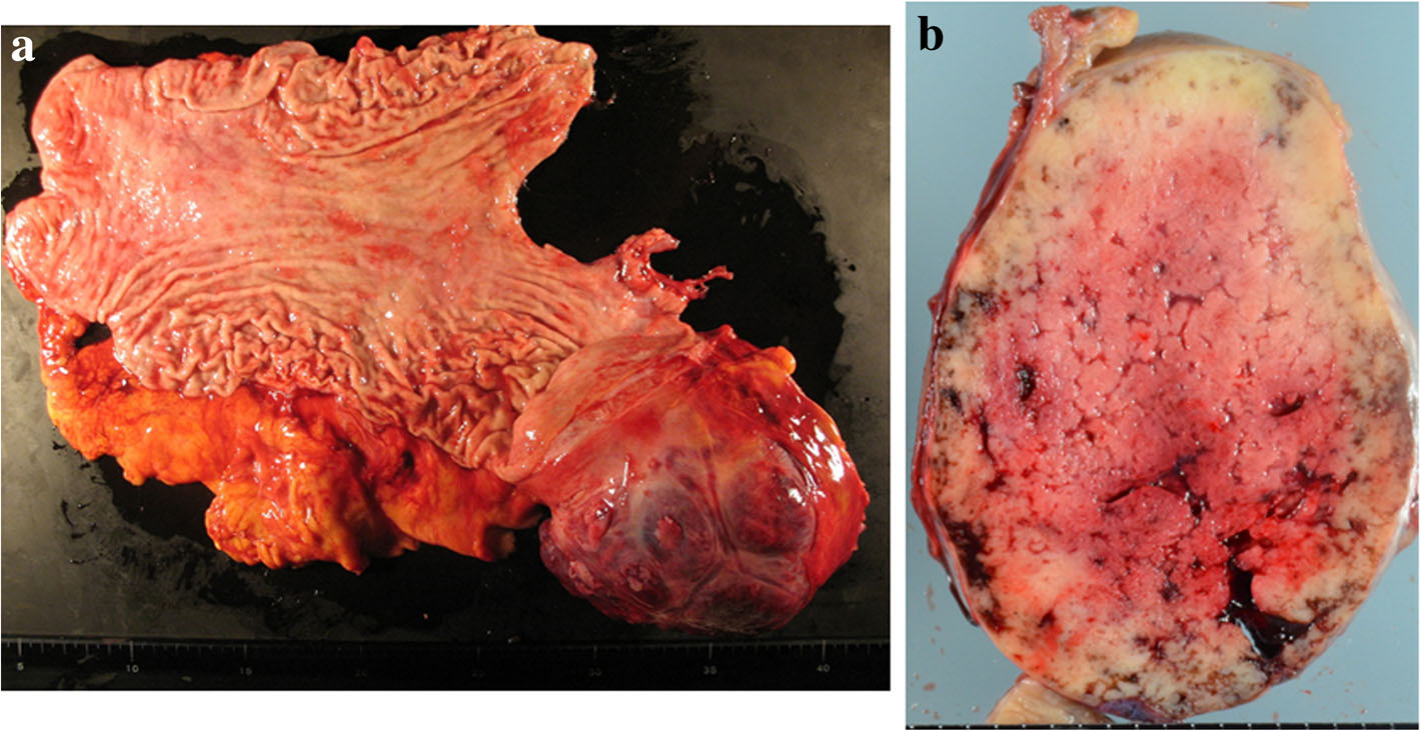
Gross examination of the resected specimen. **a** The tumor arose from the stomach wall, extended to the greater curvature of the gastric fundus, and was 140 × 135 mm in maximum diameter. **b** The cut surface of the tumor was yellowish-white and had some hemorrhagic areas, but no necrotic areas

**Fig. 6:**
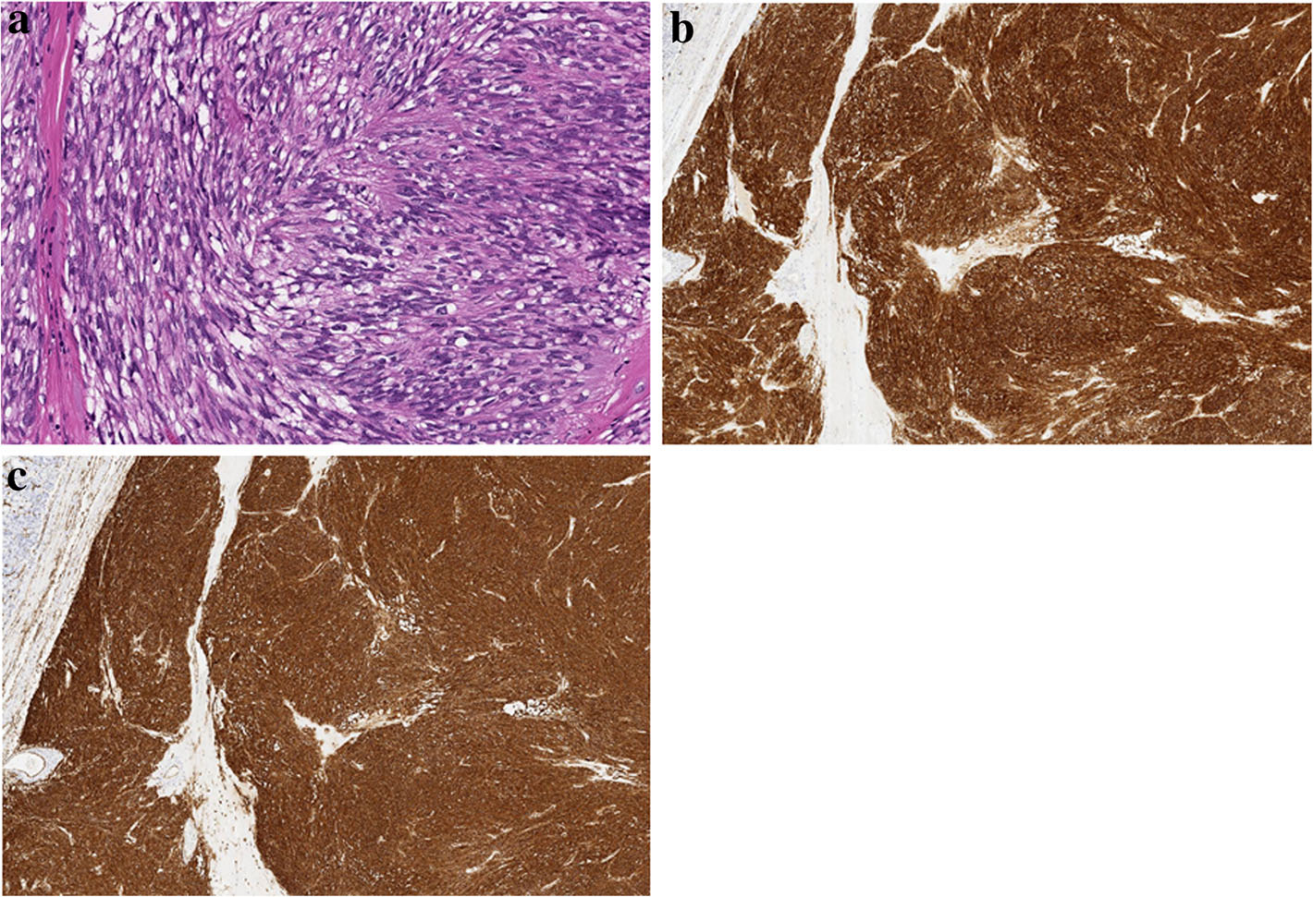
Histological findings of the resected specimen. **a** Hematoxylin and eosin staining (high-power field). **b** Positive immunostaining result for c-kit. **c** Positive immunostaining result for CD34

The resected specimen was of a tumor measuring 14.0 × 13.5 cm in maximal diameter and arising from the greater curvature of the gastric fundus (Fig. 5a). The cut surface was yellowish-white and had some hemorrhagic areas, but no necrotic areas were discovered (Fig. 5b). The histopathological examination revealed that the inside of the tumor comprised spindle cells with high nuclear-cytoplasm ratios and hyperchromasia (Fig. 6a). The mitotic index was 2 per 50 high-power fields. Through immunostaining, the tumor cells were found to be positive for CD34 and c-kit (Fig. 6b, c), but negative for S100 and SMA. Therefore, the final diagnosis was of a GIST.

The patient recovered uneventfully and was discharged from the hospital 13 days after surgery having experienced no complications. We did not provide adjuvant chemotherapy with imatinib, at the patient’s request, who cited her advanced age as the main reason for refusal. However, we continued to offer follow-up, and 24 months after surgery, she was still alive and had remained disease-free.

## Discussion

Although small GISTs are usually asymptomatic, patients may develop abdominal pain or discomfort, a palpable abdominal mass, or gastrointestinal hemorrhage if the tumors grow sufficiently large [3]. Because of the size of this particular GIST, our patient had chest discomfort. Regarding the development of the tumor, two possibilities were considered: first, that an esophageal hiatal hernia had developed and the GIST arose from the stomach and grew in the mediastinum and, second, that the GIST arose from the stomach in the abdominal cavity slid into the mediastinum because of the esophageal hiatal hernia and grew in the mediastinum. The differential diagnosis of the mediastinal tumor was a thymoma or an esophageal GIST [7–10]. However, CT showed that the tumor arose from the gastric wall and had low-density areas that suggested possible necrosis, indicating that the tumor was probably a GIST. Because of these preoperative findings, we did not attempt preoperative diagnosis using endoscopic ultrasonography-guided fine-needle aspiration. According to clinical GIST guidelines in Japan, intestinal submucosal tumors should be resected if they are symptomatic or, if asymptomatic, the tumors exceed 5.1 cm in diameter [11]. Because the tumor diameter in our patient was both larger than 5.1 cm and causing symptoms, we performed surgery.

Laparoscopic resection of GIST has become increasingly common. However, laparoscopic surgery was not suitable in this case because of the tumor’s huge size and the risk of capsule injury. If the tumor had not adhered to adjacent organs, it might have been possible to resect it through laparotomy. However, because the tumor adhered to the lower lobe of the left lung, the mediastinal pleura, and the diaphragm, we performed additional left thoracotomy. We were able to perform complete resection by thoracolaparotomy without disrupting the tumor. Partial gastrectomy was difficult because the tumor arose from the stomach wall, extended to the greater curvature of the gastric fundus, and adhered to the lower esophagus. If abdominal esophagus cannot be preserved, we do not perform proximal gastrectomy. In addition, elderly patient has a risk for aspiration because of gastroesophageal reflex after a proximal gastrectomy. In our patient, a total gastrectomy and a lower esophagectomy were appropriate.

To better understand this case, we searched the literature in PubMed and in the Japan Medical Abstracts Society database from 2002 to 2018 for the key terms “gastric GIST,” “esophageal hiatal hernia,” “GIST,” and “mediastinal.” Seven other cases of GIST associated with hiatal hernia have been reported [12–18], and we summarize the characteristics and treatment details of those seven cases and ours in Table 1. The included patients had a mean age of 74.1 years (range, 52–88), seven of the eight patients (87.5%) were female, and tumors measured 4.5–20 cm in diameter. Of the seven patients who underwent resection, two underwent total gastrectomy (28.6%), and in both those cases, the tumors were huge (14 cm in maximal diameter). In another case, total gastrectomy was performed via laparotomy, but the tumor was disrupted during the operative maneuver. Care is needed in handling a GIST to avoid disrupting the outer tumor membrane. We safely performed complete resection of a huge tumor by thoracolaparotomy, without causing tumor disruption.

**Table 1:** Reports of gastric GIST with hiatal hernia

Case	Author	Gender	Age	Chief complaint	Tumor size (cm)	Preoperative diagnosis	Operative method	Immunohistological findings	MI	Miettinen’s criteria [19]	Joensuu’s criteria [20]	Prognosis
1	Miyauchi et al. [12]	F	85	Vomiting	9	GIST	Unresectable (stent implantation)	c-kit (+), CD34 (+), Vimentin (+)	10	High risk	Moderate risk	Not described
2	Machishi et al. [13]	F	61	Anorexia and back pain	20	Posterior mediastinal tumor (suspected leiomyosarcoma)	Gastrectomy and lower esophagectomy and partial resection of lower lobe of lung	c-kit (+), CD34 (+), S-100 (+), NSE (+)	Less than 1 per 10 high power fields	Moderate risk	High risk	No recurrence after 25 months
3	Higashi et al. [14]	F	88	Dysphagia	14	SMT and adenocarcinoma	Total gastrectomy	c-kit (+), CD34 (+), SMA focal (+)	0–2 per high power field	High risk	High risk	Metastasis after 11 months
4	Kim et al. [15]	F	71	Chest pain	10	Not described	Tumorectomy and wedge resection of right lower lobe	c-kit (+), CD34 (+)	14	High risk	High risk	No recurrence after 5 years
5	Sugimoto et al. [16]	M	52	Physical fatigue and anorexia	10.5	SMT	Partial gastrectomy	c-kit (+), CD34 (+)	5	Moderate risk	High risk	Not described
6	Shiozaki et al. [17]	F	87	Nausea	4.5	SMT (suspected GIST)	Distal gastrectomy	c-kit (+), CD34 (+)	7	Moderate risk	Moderate risk	Not described
7	Yin et al. [18]	F	68	Dysphagia	13	GIST	Partial gastrectomy and lower esophagectomy	c-kit (+), CD34 (+), DOG-1 (+)	18	High risk	High risk	No recurrence after 48 months
8	Our case	F	81	Chest discomfort	14	SMT	Total gastrectomy and lower esophagectomy	c-kit (+), CD34 (+), vimentin (+)	2	Moderate risk	High risk	No recurrence after 24 months

*F* female, *GIST* gastrointestinal stromal tumor, *M* male, *MI* mitotic index (per 50 high-power fields), *SMT* submucosal tumor

Only two (25%) of the eight tumors were diagnosed before treatment. Pathological study revealed positivity for c-kit and CD34 in all cases. Risk for recurrence was high in 50% of the patients, according to Miettinen’s criteria, and in 75% according to Joensuu’s criteria [19, 20]. It was difficult to conclude whether these tumors tend to become malignant in all cases or whether tumor malignancy increased after growth in the mediastinum.

Several studies have shown that neoadjuvant chemotherapy is useful for treating primary or secondary GIST [21, 22]. We consider neoadjuvant chemotherapy for cases in which a GIST has invaded adjacent organs. In this patient, however, the tumor showed no evidence of invasion preoperatively; therefore, neoadjuvant chemotherapy was not administered preoperatively.

Immunohistochemical examination revealed a c-kit-positive tumor with a mitotic index of 2 per 50 high-power fields and a diameter of 14.0 cm at its largest aspect. Thus, the tumor was classified as having moderate risk of recurrence, according to Miettinen’s criteria, [19] and at high risk according to Joensuu’s criteria [20]. Although adjuvant chemotherapy with imatinib has been reported to be efficacious [23], our patient decided against it, citing her advanced age as the main reason.

## Conclusions

We have reported our experience with a rare case of GIST located in the mediastinum as a result of an esophageal hiatal hernia. This diagnosis should be considered in the differential diagnosis of tumors growing in the mediastinum.

## Abbreviations

CT: Computed tomography
GIST: Gastrointestinal stromal tumor
